# Bilirubin Targeting WNK1 to Alleviate NLRP3‐Mediated Neuroinflammation

**DOI:** 10.1002/advs.202407349

**Published:** 2025-03-20

**Authors:** Linfei Mao, Jiayu Lu, Quanjun Yang, Zhenqi Liu, Cuiping Wu, Bingbing Ke, Kaiyan Su, Haolin Yuan, Yaqi Cui, Yao Wang, Richard Salvi, Guang Yang, Shankai Yin, Feng Liu, Chunyan Li

**Affiliations:** ^1^ Shanghai Key Laboratory of Sleep Disordered Breathing Department of Otolaryngology‐Head and Neck Surgery Otolaryngology Institute of Shanghai Jiao Tong University Shanghai Sixth People's Hospital Affiliated to Shanghai Jiao Tong University School of Medicine Shanghai 200233 China; ^2^ Department of Stomatology Shanghai Jiao Tong University Affiliated Sixth People's Hospital Shanghai 200233 China; ^3^ Department of Pharmacy Shanghai Sixth People's Hospital Affiliated to Shanghai Jiao Tong University School of Medicine Shanghai 200233 China; ^4^ Center for Hearing and Deafness State University of New York at Buffalo Buffalo NY 14214 USA

**Keywords:** bilirubin, neuroinflammation, NLRP3, phosphorylation, WNK kinase

## Abstract

Bilirubin, an endogenous metabolite with many significant physiological roles, particularly anti‐inflammatory properties, shows great promise as a treatment for inflammatory diseases. However, the binding targets and downstream signaling mechanisms of bilirubin remain unclear. Here, by using quantitative phosphorylation proteomics and several powerful chemical biology techniques such as the Cellular Thermal Shift Assay (CETSA), molecular docking, and MicroScale Thermophoresis (MST), it is identified and confirmed that with‐no‐lysine (K) kinase 1 (WNK1) is the primary target of bilirubin at physiological concentrations. Bilirubin binds to the kinase domain of WNK1, activating its kinase activity and increasing the intracellular chloride ion concentration via the downstream SPAK/OSR1‐KCC2 pathway in neurons. Manipulating endogenous bilirubin levels by deleting *Blvra*, the bilirubin synthesis enzyme, and *Ugt1a1*, its metabolic enzyme, significantly promotes and inhibits the activation of the lipopolysaccharide (LPS)‐induced NLRP3 inflammasome, respectively, in mouse hippocampus. Similarly, exogenous bilirubin supplementation suppressed LPS‐induced NLRP3 inflammasome activation in mouse hippocampus in a WNK1‐dependent manner. Quantitative phosphoproteomic analysis of WNK1 downstream signaling elucidated the broad biological roles of WNK1, notably its function in suppressing inflammation. The findings clarify the direct targets and signaling mechanisms underlying the anti‐inflammatory effects of bilirubin and pave the way for exploring its novel functions.

## Graphical abstract

At physiological concentrations, bilirubin binds to the kinase domain of WNK1, thereby augmenting its activity and facilitating the phosphorylation of downstream SPAK/OSR1. This phosphorylation inhibits KCC2 activity, leading to elevate intracellular chloride levels in neurons. Moreover, the interaction between bilirubin and WNK1 suppresses the activation of LPS‐induced NLRP3 inflammasomes, thereby exerting anti‐inflammatory effects.

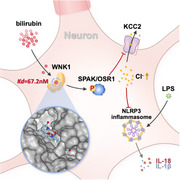

## 1 Introduction

Bilirubin, a heme metabolite produced during the hepatic processing of hemoglobin, was once viewed merely as a metabolic waste product or a cause of brain damage in neonates when present in excess.^[^ ^1^ ^]^ However, recent studies have recognized its significant physiological functions.^[^ ^2^ ^]^ Emerging clinical evidence indicates that bilirubin is a crucial signaling molecule involved in the pathophysiology of numerous immune, infectious, and metabolic diseases. Even minor decreases in serum bilirubin levels are associated with an increased risk for these conditions.^[^ ^3^ ^]^ Furthermore, various synthesized nanoparticles have been developed to elevate bilirubin levels for the treatment of inflammatory and autoimmune diseases in clinical trials.^[^ ^4^ ^]^ The beneficial effects of bilirubin are attributed to its versatile functions, including hormone mimicry, clearance of oxygen free radicals, and suppression of nearly all immune system effectors.^[^ ^5^ ^]^ The physiological function of bilirubin is presumed to result from inhibition of protein phosphorylation and activation of various nuclear and cytoplasmic receptors.^[^ ^6^ ^]^ However, the molecular mechanisms by which bilirubin affects cell signaling and contributes to its physiological functions remain poorly understood.

In the bloodstream, most bilirubin is bound to albumin, which facilitates stable circulation. Free bilirubin, being lipophilic, can cross both peripheral and central physiological barriers and cell membranes.^[^ ^7^ ^]^ As a signaling molecule, bilirubin is believed to influence various cellular regulatory systems by binding to cell membranes and intracellular target proteins, thereby modulating subsequent cell signaling.^[^ ^8^ ^]^ Identifying these target proteins is crucial for fully understanding the molecular mechanisms underlying bilirubin's extensive biological effects. In the peripheral system, bilirubin interacts with several intracellular and nuclear receptors, such as peroxisome proliferator‐activated receptors (PPAR) and liver‐specific fatty acid binding protein (FABP1), modulating various metabolic pathways.^[^ ^3^ ^]^ At pathological high levels, bilirubin can bind to Mas‐related G‐protein coupled receptors (MRGPRs) to induce cholestatic itch.^[^ ^9^ ^]^ Notably, bilirubin plays a particularly significant role in the central nervous system, where it is involved in signaling pathways that counteract inflammation associated with chronic neurodegenerative, neuropsychiatric, and other neurological diseases. However, severe elevation of bilirubin can lead to or exacerbate neural injury. We have previously reported that elevated serum bilirubin levels in patients with stroke directly activate the transient receptor potential melastatin 2 (TRPM2) channel on neuron cell membranes, worsening brain damage.^[^ ^10^ ^]^ Despite these findings, the precise target proteins through which bilirubin exerts its cellular regulatory functions and contributes to its anti‐inflammatory roles remain unclear.

Bilirubin exerts widespread inhibitory effects on protein phosphorylation, significantly modulating intracellular signaling pathways across various biological processes.^[^ ^6^, ^11^ ^]^ For instance, bilirubin alleviates group 2 innate lymphoid cells (ILC2) driven airway inflammation by downregulating the phosphorylation of extracellular signal‐regulated kinase (ERK)1/2.^[^ ^12^ ^]^ Protein phosphorylation is crucial for modulating protein function and controlling inflammatory, immune, and metabolic processes, such as NOD‐like receptor thermal protein domain‐associated protein 3 (NLRP3) inflammasome assembly and activation.^[^ ^13^, ^14^, ^15^ ^]^ These findings prompted us to investigate the physiological protein targets of bilirubin by analyzing its phosphorylation regulatory network.

In this study, we conducted quantitative phosphorylation proteomics to analyze the kinase‐substrate network modulated by bilirubin in the cochlear nucleus. We utilized a combination of the Cellular Thermal Shift Assay (CETSA), molecular docking, and MicroScale Thermophoresis (MST) to identify the targets of bilirubin and the binding sites among differentially phosphorylated proteins. Using these robust techniques, we determined that with‐no‐lysine (K) kinase 1 (WNK1) binds directly to bilirubin at physiological concentrations. WNK1, a ubiquitously expressed serine‐threonine kinase, is part of a signaling cascade that is essential for chloride ion homeostasis, immune regulation, autophagy, and other processes.^[^ ^16^, ^17^ ^]^ Our results demonstrated that bilirubin has a strong direct interaction with the kinase domain of WNK1, promoting its kinase activity and elevating intracellular chloride ion levels in neurons. Furthermore, we examined the effects of bilirubin on WNK1 in vivo and in vitro, identifying an essential role for WNK1 in LPS‐induced inactivation of the NLRP3 inflammasome through the activation of WNK1 kinase function.

## 2 Results

### 2.1 Integrative Analysis of Phosphoproteomics and CETSA Identifies WNK1 as a Preferred Target of Bilirubin

**Figure 1:**
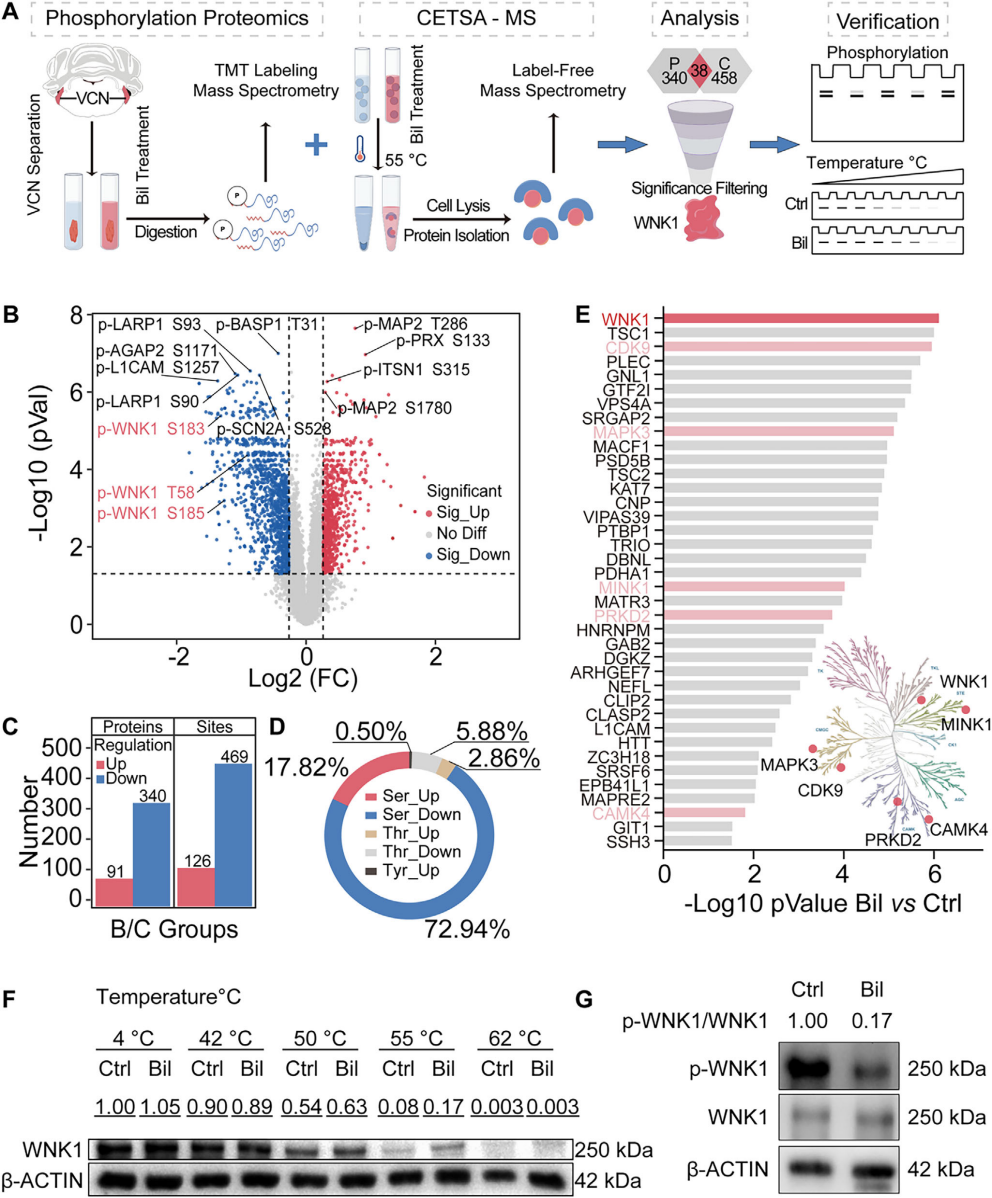
Integrative analysis of phosphorylation proteomics and CETSA to identify bilirubin's downstream signaling and binding targets. A) Workflow for identifying bilirubin targets. B) Volcano plot illustrating phosphorylation sites identified after bilirubin treatment. C) Number of significantly regulated proteins and phosphorylation sites following bilirubin treatment. D) Distribution of significantly altered phosphorylation site amino acid types. E) Distribution of top candidate targets matching bilirubin from the integrated analysis of two proteomics datasets. Protein kinases (pink and red) are labeled and mapped onto the human kinase spectrum (bottom right), with WNK1, the most significantly associated kinase target, highlighted in red. F) CETSA‐Western blotting experiment confirms the interaction between bilirubin and WNK1. G) Western blotting analysis of WNK1 Thr60 phosphorylation in control and bilirubin‐treated mouse cochlear nuclei for 30 min. In panels B–D), all bilirubin treatment groups were compared with their respective control groups. Significantly regulated phosphorylation sites were defined as those having an absolute value of bilirubin/control >1.5 and an adjusted *p* < 0.05. See also Figure S1 (Supporting Information).

To elucidate the direct binding targets and downstream signaling pathways of bilirubin, we conducted quantitative phosphoproteomic and drug‐protein binding studies, followed by integrated analysis. Both human and animal studies have demonstrated that bilirubin preferentially deposits in specific brain regions, which are recognized as bilirubin‐sensitive, including the brainstem nuclei (e.g., cochlear nucleus, vestibular nuclei, superior olivary complex), hippocampus and cerebellum. Bilirubin‐sensitive cochlear nuclei from mice were incubated in artificial cerebrospinal fluid (ACSF) with or without 3 µM bilirubin (**Figure** **1**A).^[^ ^18^ ^]^ Tandem mass tag (TMT)‐based phosphoproteomic methods were used to assess changes in protein abundance and phosphorylation at the whole‐proteome level (Figure 1A).^[^ ^19^, ^20^ ^]^ Principal component analysis (PCA), Pearson correlation coefficients, and relative standard deviations (RSD) indicated significant differences between bilirubin‐treated and control samples (Figure S1A–C, Supporting Information). We quantified 11116 phosphorylation sites across the 3474 proteins (Figure 1B). Bilirubin treatment resulted in a notable reduction in significantly regulated phosphorylation sites and proteins, with 340 proteins showing decreased phosphorylation at 469 sites and 91 proteins showing increased phosphorylation at 126 sites (Figure 1C). These modifications predominantly affected serine/threonine residues, with 75.8% involving phosphorylation and 17.82% dephosphorylation of serine residues (Figure 1D). Motif analysis identified the significant motif xxxxPx_S_PxxxRx (Figure S2A–C, Supporting Information). Enrichment analysis revealed that the phosphorylated proteins responsive to bilirubin were mainly associated with neurodevelopment, neurogenesis, autophagy, and synaptic structures (Figure S1D,E, Supporting Information).

Protein kinases are key regulators of cell signaling mediated by reversible phosphorylation that relies on protein–protein binding between kinases and their regulators. Protein kinase analysis identified several kinase families, including mitogen‐activated protein kinase (MAPK) and WNK, that were significantly affected by bilirubin (Figure S1F, Supporting Information). Notably, the WNK family members exhibited the most significant decrease in phosphorylation levels following bilirubin treatment (Figure S1G, Supporting Information).

To further clarify the potential binding targets of bilirubin, we used a label‐free technique based on protein thermal stability, known as CETSA, combined with mass spectrometry (MS). This method is widely applicable for exploring protein‐molecule interactions.^[^ ^21^, ^22^ ^]^ SHSY‐5Y cell lysates were incubated with bilirubin, followed by denaturation at 55 °C. Proteins bound to bilirubin exhibited altered solubility owing to changes in thermal stability (Figure 1A). MS analysis of the soluble fraction revealed an increase in the abundance of 458 proteins and a decrease in that of 149 proteins, indicating their direct or indirect association with bilirubin (Figure S3, Supporting Information). Functional annotation and enrichment analysis of proteins with increased solubility highlighted an overrepresentation of proteins involved in the “cellular redox homeostasis” pathway, aligning with the predicted function of bilirubin as a redox reagent. Additionally, proteins related to “UDP‐glucosyltransferase activity,” a pathway directly linked to bilirubin metabolism, were identified. Conversely, proteins with decreased solubility were significantly enriched in the “peroxisome” pathway, consistent with studies showing that bilirubin modulates peroxisome proliferator‐activated receptor alpha (PPARα) activity.^[^ ^23^ ^]^ Notably, the “protein serine/threonine kinase activity” pathway was also enriched among proteins with increased solubility (Figure S4A,B, Supporting Information).

Integrated analysis of phosphoproteomic and binding proteomic data has identified 38 proteins as candidate bilirubin‐binding targets that also affect phosphorylation. Among these, WNK1 emerged as the most promising candidate, with a *p*‐value of 7.68 × 10^−7^ (Figure 1E; Figure S5A, Supporting Information). Further analysis using the Allen Brain Atlas and immunofluorescence staining revealed that WNK1 is highly expressed in bilirubin‐sensitive brain regions, such as the hippocampus and cochlear nucleus (Figure S5B–E, Supporting Information). Additionally, domain enrichment analysis of downregulated phosphorylated proteins after bilirubin treatment highlighted the “Serine/threonine‐protein kinase OSR1/WNK, CCT domain” (Figure S6A,B, Supporting Information). These results suggest that WNK1 is a potential target for bilirubin in the brain.

To validate these findings, SH‐SY5Y cells were incubated in a serum‐free medium with bilirubin (6 µM) or an equivalent amount of DMSO as the control, followed by protein denaturation at different temperatures (Figure 1A). Western blotting analysis of the soluble fraction showed an enhanced thermal stability of WNK1 in the bilirubin‐treated group (Figure 1F). Phosphoproteomic analysis revealed a significant decrease in WNK1 phosphorylation at Thr58 (corresponding to Thr60 in humans), Ser183, and Ser185 (Figure 1B; Figure S7A–D, Supporting Information). Western blotting results further demonstrated a substantial reduction in WNK1 phosphorylation at Thr58 (corresponding to Thr60 in humans) in the cochlear nucleus after bilirubin treatment (Figure 1G). These results strongly suggest that WNK1 is a target of bilirubin.

### 2.2 Bilirubin Directly Binds to WNK1

**Figure 2:**
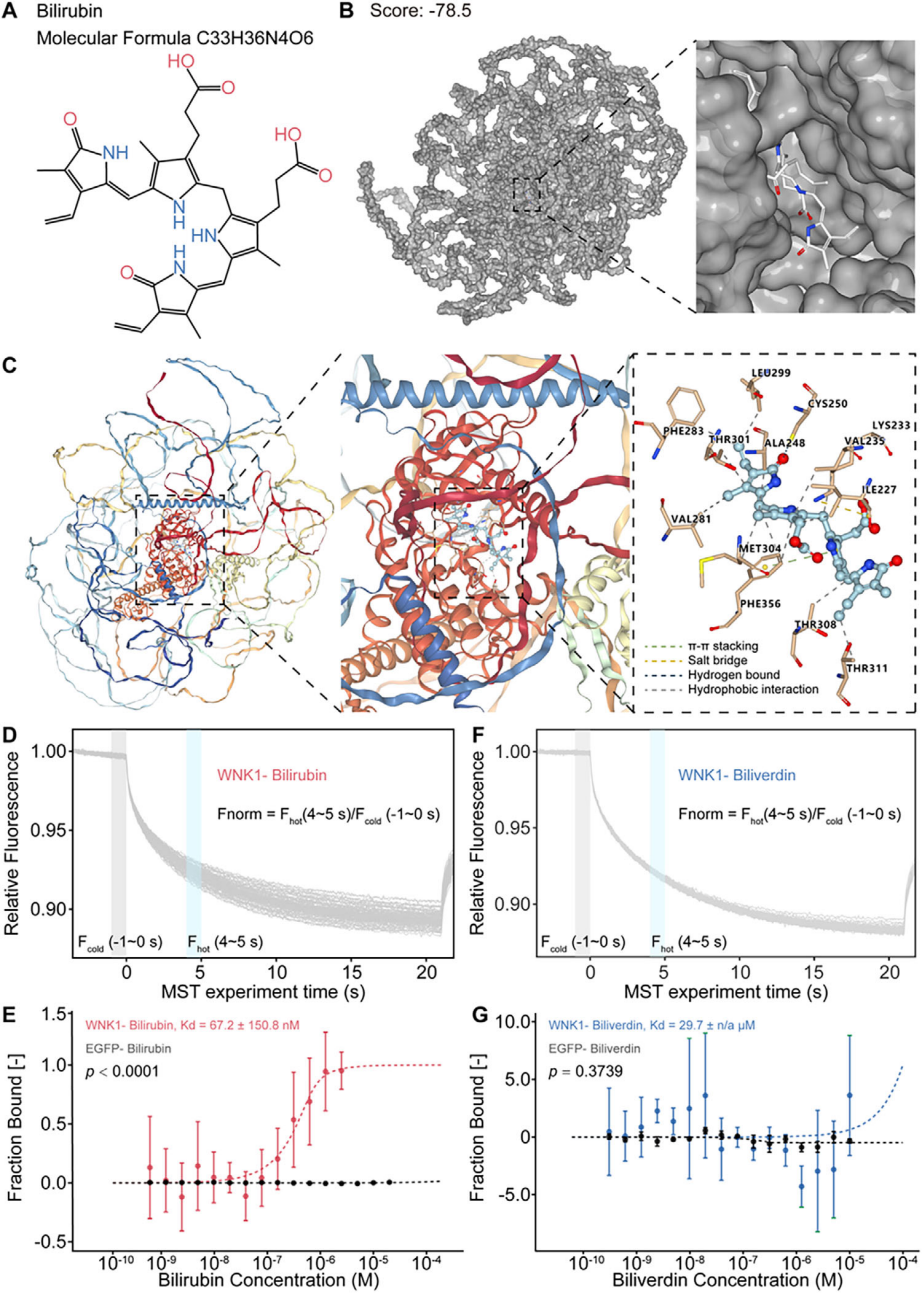
Bilirubin directly binds to WNK1. A) Chemical structure and molecular formula of bilirubin. B) Molecular docking showing an overview of bilirubin‐bound hWNK1 (left panel) and a close‐up view of the bilirubin‐binding site (right panel). The grid score for bilirubin‐bound hWNK1 is shown. C) Specific recognition of bilirubin‐bound hWNK1. 3D diagram showing bilirubin binding to the deep cavity of the WNK1 protein kinase domain, and a 2D diagram illustrating the interactions through hydrogen bonds, salt bridges, π–π interactions, and hydrophobic interactions. D) MST experimental traces of full‐length hWNK1 with bilirubin, with vertical bars indicating fluorescence changes (gray cold; blue hot). A formula for calculating Fnorm is also presented. E) MST demonstrating a direct interaction between bilirubin and EGFP‐tagged WNK1 (15 min) in lysates from EGFP‐WNK1 expressing HEK293T cells (*n* = 6 per group). F) MST experimental traces of full‐length hWNK1 with biliverdin, with vertical bars indicating fluorescence changes (gray cold; blue hot). A formula for calculating Fnorm is also presented. G) MST demonstrating a direct interaction between biliverdin and EGFP‐tagged WNK1 (15 min) in lysates from EGFP‐WNK1 expressing HEK293T cells (*n* = 3 per group). In panels E) and G), EGFP in lysates from EGFP‐expressing HEK293T cells was co‐incubated with either bilirubin or biliverdin for 15 min as a control (*n* = 3/group). Error bars indicate mean ± SD.

To investigate the direct interaction between bilirubin and WNK1, we initially obtained a three‐dimensional structure of WNK1 from *Alphafold2*.^[^ ^24^ ^]^ Subsequently, we performed molecular docking between the crystal structure of bilirubin and WNK1 using *DOCK 6.9* application (**Figure** **2**A–C).^[^ ^25^ ^]^ The results revealed a high affinity between bilirubin and the 195–483 region of human WNK1 (hWNK1, Score = −78.5), which corresponds to the catalytic domain of WNK1 (Figure 2B). The detailed analysis identified multiple residues within this region that involved hydrophobic interactions (Ile227, Lys233, Val235, Ala248, Val281, Phe283, Leu299, Thr301, Met304, Thr308, Thr311, and Phe356), salt bridges (Lys233), π‐π stacking (Phe356), and hydrogen bonds (Cys250) with bilirubin. Notably, several of these residues were active sites for the WNK1 kinase domain and ATP binding sites (Ile227, Lys233, Val235, Ala248, Cys250, Val281, Thr301, Met304, Thr308, and Phe356) (Figure 2C).

To validate the direct binding of bilirubin to WNK1, we performed a Microscale Thermophoresis (MST) analysis, which assesses the binding capacity of a compound by measuring the movement of proteins over a microscopic temperature gradient.^[^ ^26^, ^27^ ^]^ We overexpressed full‐length WNK1 fused with Green Fluorescent Protein (GFP) in HEK‐293 cells, incubated the lysates with bilirubin at varying dilutions, and subjected them to precise MST measurements in capillaries. This approach conclusively demonstrated a direct interaction between bilirubin and full‐length hWNK1, with a strong binding affinity (*Kd* = 67.2 nM, *p* < 0.0001) (Figure 2D,E). This binding concentration is significantly lower than the concentrations at which bilirubin exerts toxic effects in the central nervous system and is comparable to its physiological concentration in vivo. Furthermore, biliverdin, a structurally similar intermediate product generated before bilirubin formation, was used as a control. Biliverdin showed minimal binding (*Kd* = 29.7 µM, *p* = 0.3739) (Figure 2F,G). Additionally, compared to other members of the WNK kinase family, WNK1 exhibited a significantly higher binding affinity to bilirubin (*Kd* = 0.53 µM for hWNK2; *Kd* = 0.76 µM for hWNK3; *Kd* = 0.28 µM for hWNK4) (Figure S8A, Supporting Information). Collectively, these findings indicated that WNK1 is a physiological target of bilirubin.

### 2.3 Bilirubin Directly Binds to the WNK1 Kinase Domain

**Figure 3:**
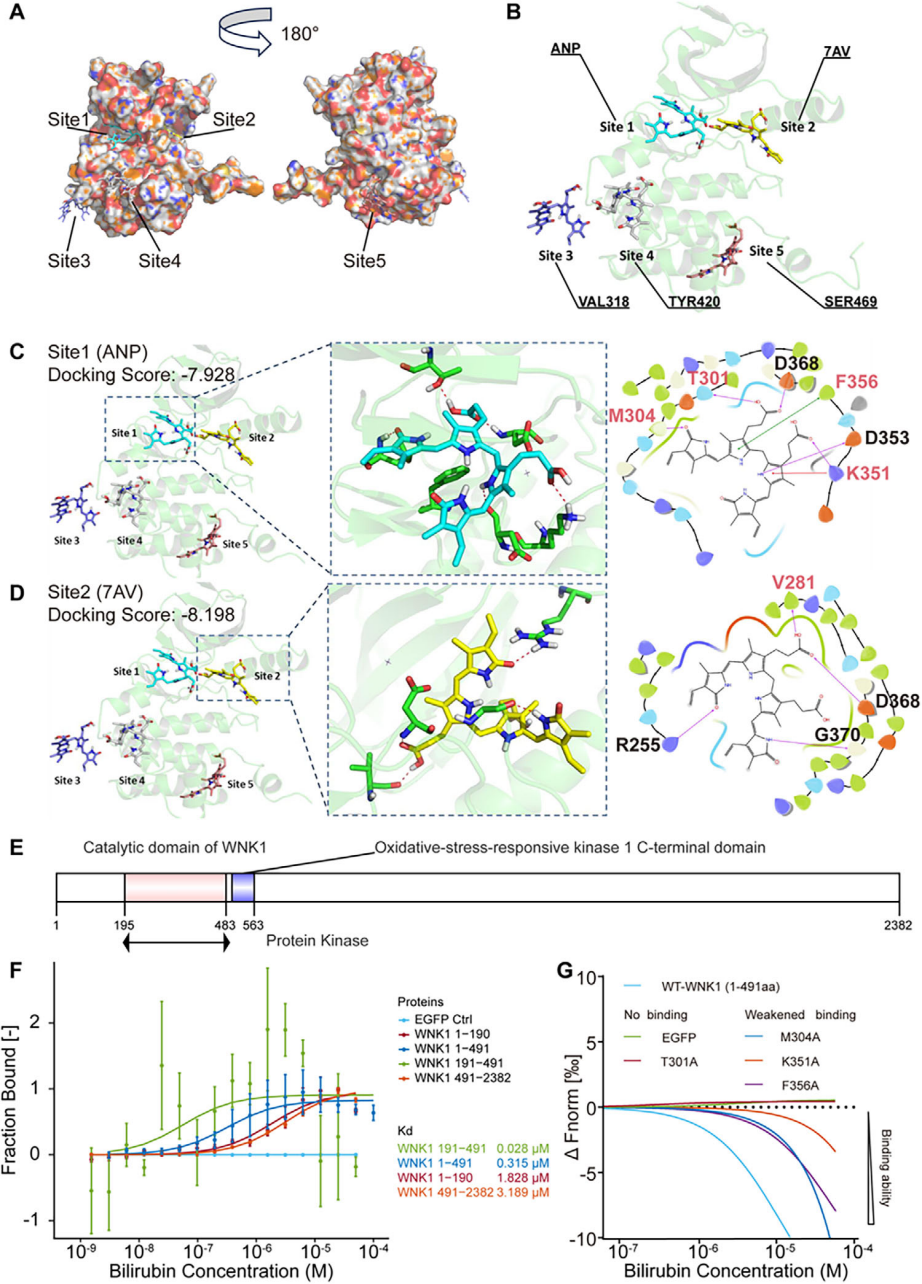
Bilirubin binds to the WNK1 kinase domain. A) Interaction surface diagram of bilirubin with the hWNK1 kinase domain crystal structure (PDB ID 5TF9). B) Cartoon diagram illustrating the interaction of bilirubin with the hWNK1 kinase domain crystal structure, highlighting the binding pocket. C) Specific recognition of the bilirubin‐bound hWNK1 ATP‐binding pocket. D) Specific recognition of the bilirubin‐bound hWNK1 ligand‐binding pocket. E) Schematic representation of the hWNK1 protein domains. F) MST demonstrating a direct interaction between bilirubin and EGFP‐tagged WNK1 (1–190), WNK1 (191–491), and WNK1 (491–2382) (15 min) in lysates from different domains of EGFP‐WNK1 expressing HEK293T cells (*n* = 3/group). G) MST demonstrating a direct interaction between bilirubin and EGFP‐tagged point mutations of WNK1 (1–491) (15 min) in lysates from different plasmids expressing HEK293T cells. In panels F) and G), EGFP in lysates from EGFP‐expressing HEK293T cells was co‐incubated with bilirubin for 15 min as a control (*n* = 3/group). Error bars indicate mean ± SD.

We hypothesized that the kinase domain of WNK1 is a preferred binding region for bilirubin. Given the availability of the crystal structure of the N‐terminal region (206–483) of hWNK1 (hWNK1‐N), which contains the kinase domain, we conducted molecular docking analyses to investigate the binding of bilirubin to hWNK1‐N.^[^ ^28^ ^]^ The *Sitemap* module predicted five potential bilirubin‐binding pockets within the hWNK1‐N region, including the ATP‐binding pocket centered on ANP, the ligand‐binding pocket centered on the ligand molecule 7AV, and binding pockets centered on Val318, Tyr420, and Ser469 (**Figure** **3**A,B). The results demonstrate that bilirubin exhibited the highest affinity for the ATP‐ and substrate‐binding domains of hWNK1, with docking scores of −7.928 and −8.198, respectively (Figure 3C,D). In contrast, the scores for Sites 3–5 were relatively lower, at −2.729, −1.787, and −3.526, respectively (Figure S9A–C, Supporting Information).

To further validate these findings, we constructed GFP‐tagged fusion proteins with different lengths of WNK1 truncations (1–491, 1–190, 191–491, 491–2382) based on the WNK1 domains (Figure 3E) and analyzed their binding capacity with bilirubin using MST analysis.^[^ ^29^ ^]^ The results indicated that the fragments containing the kinase domain of WNK1 (1–491 and 191–491) exhibited significantly higher binding affinities with bilirubin (*Kd* = 0.315 and 0.028 µM, respectively) than the non‐kinase segments (*Kd* = 1.828 µM for WNK1 1–90; *Kd* = 3.189 µM for WNK1 491–2382) (Figure 3F).

Molecular docking identified several crucial amino acid residues within binding site 1 of the kinase domain for the binding of WNK1 and bilirubin. These residues included Thr301 (T301), Met304 (M304), Lys351 (K351), and Phe356 (F356) (Figure 3C). To investigate this further, we constructed WNK1‐N mutants by replacing these residues with alanine (A). The results showed that the T301A mutant was unable to bind bilirubin, whereas the M304A, K351A, and F356A mutants exhibited reduced binding affinity (Figure 3G). This finding highlights the critical role of these residues in bilirubin binding. Additionally, comparative analysis of WNK1 sequences across various mammalian species revealed that the kinase domain of WNK1 is highly conserved (Figure S8B, Supporting Information). This suggests that the interaction between WNK1 and bilirubin is a universal and evolutionarily significant feature.

### 2.4 Bilirubin Activates WNK1 Kinase to Elevate Intracellular Chloride Ion Levels

**Figure 4:**
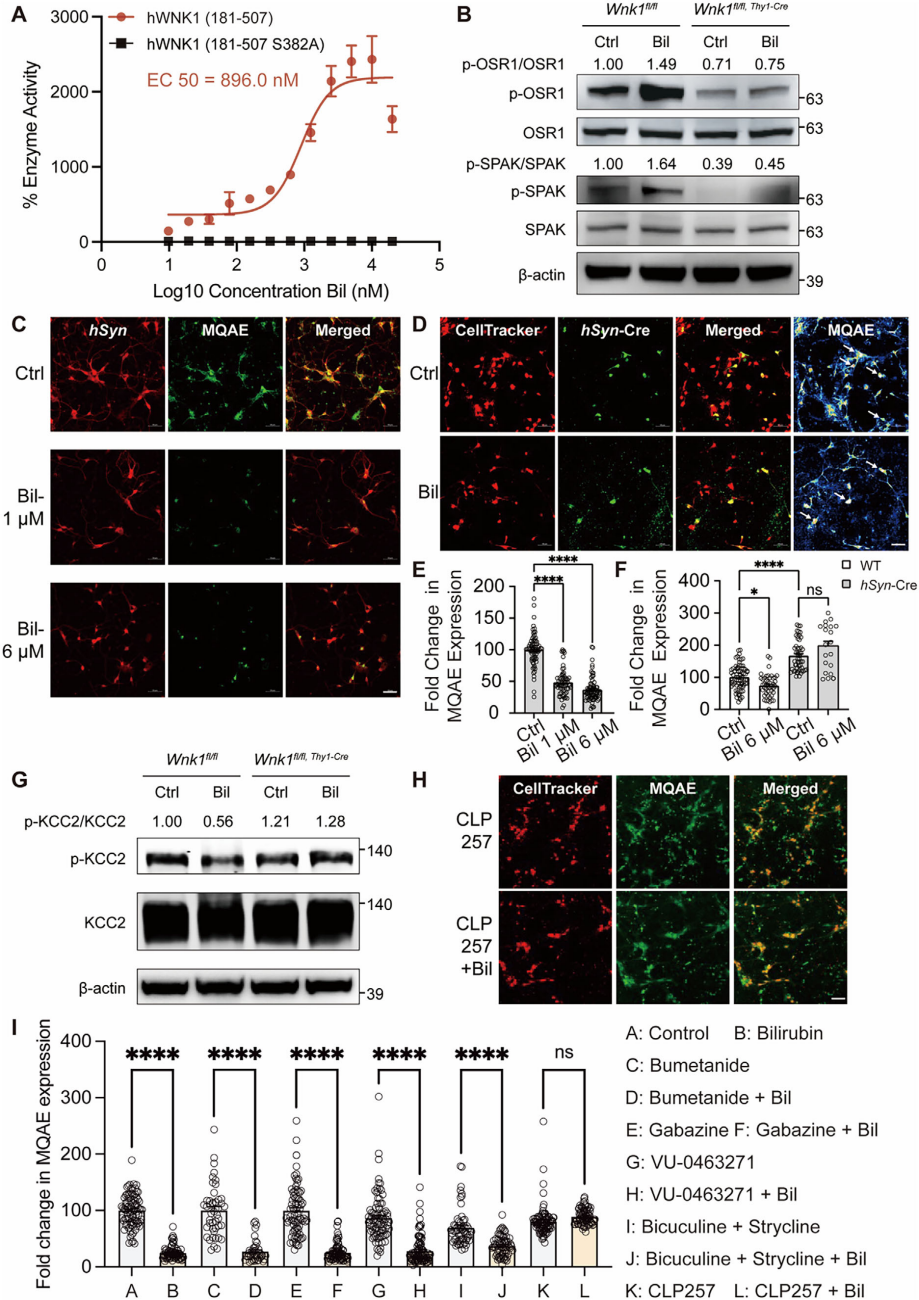
Bilirubin activates WNK1 kinase to elevate intracellular chloride ion levels via the WNK1‐KCC2 Pathway. A) Activity of the purified hWNK1 kinase domain with varying bilirubin concentrations, measured using the ADP‐Glo kinase assay. Kinase functional analysis of hWNK1 (181–507 S382A) as a control. Measured luminescence values were normalized to reactions lacking bilirubin. Data are from triplicate measurements in three independent experiments. B) Western blotting analysis of OSR1 phosphorylation (left) and SPAK phosphorylation (right) in the hippocampus of *Wnk1^fl/fl^* and *Wnk1^fl/fl,Thy1‐Cre^* mice treated with ACSF and bilirubin. C) Immunofluorescence staining of bilirubin‐induced [Cl⁻]_i_ increase in DIV8 primary cultured hippocampal neurons. MQAE staining (green) indicates [Cl⁻]_i_ (fluorescence intensity inversely proportional to intracellular chloride concentration), and *hSyn* staining (red) marks live neurons. Scale bars = 50 µm. D) Immunofluorescence staining of bilirubin‐induced [Cl⁻]_i_ increase in DIV8 primary cultured hippocampal neurons, with CellTracker staining (red) marking live cells and *hSyn*‐CRE (green) marking *Wnk1^hSyn‐cKO^* neurons. Scale bars = 50 µm. E) Summary data showing the fold‐change in MQAE fluorescence intensity of neurons treated with different bilirubin concentrations as shown in C). F) Summary data showing the fold‐change in MQAE fluorescence intensity of neurons as shown in D). G) Western blotting analysis of KCC2 phosphorylation in the hippocampus of *Wnk1^fl/fl^* and *Wnk1^fl/fl,Thy1‐Cre^* mice treated with ACSF and bilirubin. H) Immunofluorescence staining of bilirubin‐induced [Cl⁻]_i_ changes in DIV8 primary cultured hippocampal neurons, with or without CLP257. MQAE staining (green) indicates [Cl⁻]_i_, and CellTracker staining (red) marks live cells. Scale bars = 50 µm. I) Summary data showing fold‐changes in MQAE fluorescence of neurons treated with different drugs as shown in panel H) and Figure S14A (Supporting Information). Error bars indicate mean ± SD. *p < 0.05, **p < 0.01, ***p < 0.001, and ****p < 0.0001.

Given the binding of bilirubin to the kinase domain of WNK1, we aimed to explore its effect on WNK1 with a particular focus on its kinase activity. To evaluate WNK1 kinase activity, we utilized an in vitro kinase enzyme assay system to quantify ADP production by the amino acid fragment 181–507 of human WNK1 (hWNK1) over a range of bilirubin concentrations. The kinase‐dead WNK1 mutant hWNK1(181–507 S382A) served as a negative control (Figure S10, Supporting Information).^[^ ^30^ ^]^ Our findings indicate that bilirubin activates the kinase activity of WNK1, with an EC50 value of 896 nM, while showing no effect on the kinase‐dead S382A WNK1 mutant (**Figure** **4**A).

To further investigate the impact of bilirubin on WNK1 kinase activity, we generated hippocampal neuron‐specific *Wnk1* knockout mice. Given that conventional *Wnk1* knockout mice are embryonically lethal, we employed the CRISPR/Cas9 method to create mice carrying the floxed *Wnk1* gene. Then we crossed *Thy1*‐Cre mice with *Wnk1^flox/flox^* mice to generate hippocampal neuron‐specific *Wnk1* knockout mice. We examined the effects of bilirubin on the phosphorylation of WNK1 downstream targets, Ste20‐related proline alanine‐rich kinase (SPAK), and oxidative stress‐responsive kinase 1 (OSR1), in the hippocampus of *Wnk1^flox/flox^* and *Wnk1^flox/flox,Thy1‐Cre^* mice. Upon bilirubin treatment, phosphorylation of OSR1 and SPAK was elevated in the hippocampus of *Wnk1^flox/flox^* mice. However, in *Wnk1^flox/flox,Thy1‐Cre^* mice, bilirubin treatment had minimal impact on OSR1 and SPAK phosphorylation. These findings indicate that the effect of bilirubin on OSR1 and SPAK phosphorylation is WNK1‐dependent (Figure 4B). To rule out the potential effects of osmolarity on the phosphorylation of WNK1, OSR1, and SPAK, we measured the osmolarity of different bilirubin concentrations. We found that low concentrations of bilirubin didn't change the osmolarity (Figure S11, Supporting Information).

Previous studies have demonstrated that WNK1 influences the intracellular chloride ion concentration through SPAK/OSR1 by modulating chloride cotransporters, such as the Na‐K‐2Cl cotransporter NKCC1 and K‐Cl cotransporters (KCCs).^[^ ^17^, ^31^ ^]^ Therefore, we hypothesized that bilirubin activation of WNK1 kinase activity might affect intracellular chloride levels. To test this hypothesis, we treated hippocampal neurons with varying concentrations of bilirubin for 30 min and observed a significant increase in intracellular chloride concentration. This was detected using the sensitive molecular probe N‐(Ethoxycarbonylmethyl)‐6‐Methoxyquinolinium Bromide (MQAE), as indicated by a reduction in MQAE fluorescence intensity (Figure 4C,E).^[^ ^32^ ^]^

To ascertain whether bilirubin regulates chloride ions through WNK1, we generated *Wnk1* gene knockout primary cultured neurons. We achieved neuron‐specific deletion of *Wnk1* in hippocampal neurons by transducing primary neurons isolated from *Wnk1^flox/flox^* embryonic mice with a lentivirus expressing *hSyn*‐Cre‐EGFP.^[^ ^33^, ^34^ ^]^ Our results demonstrate that *Wnk1* deficiency significantly reduced intracellular chloride ion ([Cl⁻]_i_) levels in neurons. Importantly, following *Wnk1* deletion, bilirubin stimulation failed to increase [Cl⁻]_i_ levels (Figure 4D,F), indicating that WNK1 is required for bilirubin‐induced increase in [Cl⁻]_i_ levels. Intracellular chloride concentration is crucial for modulating inhibitory synaptic transmission and, consequently, neuronal excitability. Consistent with this, we found that bilirubin induces neural hyperexcitability in the cochlear nuclei and hippocampus neurons, and the WNK1 inhibitor WNK‐IN‐11 effectively suppresses bilirubin‐induced hyperexcitability (Figure S12A–D; Figure S13A–C, Supporting Information).

Since bilirubin‐induced activation of WNK1 kinase activity increases [Cl⁻]_i_ in neurons, we sought to elucidate the downstream effector proteins of WNK1 that are involved in this process. Neuronal [Cl⁻]_i_ is primarily regulated by the Cl⁻‐K⁺ symporter KCC2, with additional modulation by factors such as the Na⁺‐K⁺‐Cl⁻ co‐transporter NKCC1, GABA_A_ receptors, and glycine receptors (GlyR), all of which contribute to [Cl⁻]_i_ homeostasis.^[^ ^31^ ^]^ To investigate the mechanism underlying bilirubin‐induced changes in [Cl⁻]_i_, we selectively targeted these proteins using pharmacological inhibitors (VU0463271 for KCC2, bumetanide for NKCC1, bicuculline for GABA_A_ receptors, and strychnine for GlyR) and activated KCC2 using CLP257.^[^ ^35^, ^36^ ^]^ Consistent with the role of KCC2 as a key regulator of Cl⁻ efflux in neurons, activation of KCC2 with CLP257 counteracted the effect of bilirubin on [Cl⁻]_i_, preventing its elevation (Figure 4H). Conversely, other pharmacological agents did not produce similar effects (Figure 4I; Figure S14A, Supporting Information). Additionally, we observed a decrease in the relative expression of phospho‐S940 KCC2, a phosphorylation event known to enhance KCC2 activity, in *Wnk1^fl/fl^* mice hippocampal tissue following bilirubin treatment, whereas no such effect was observed in *Wnk1^fl/fl,Thy1‐Cre^* mice (Figure 4G).^[^ ^37^ ^]^ The bilirubin‐induced effect on [Cl⁻]_i_ was not observed in primary astrocytes (Figure S15A,B, Supporting Information) or the HEK 293T cell line (Figure S15C, Supporting Information), both of which lacked KCC2 expression (Figure S16A,B, Supporting Information), further confirming that the effects of bilirubin and WNK1 on [Cl⁻]_i_ elevation are dependent on KCC2. These findings suggest that KCC2 significantly contributes to the elevation in [Cl⁻]_i_ of neurons following bilirubin binding to WNK1.

### 2.5 Bilirubin‐WNK1 Binding Suppresses LPS‐Induced Neuronal NLRP3 Activation

The physiological concentration of bilirubin exerts broad anti‐inflammatory effects. Previous studies have reported that WNK1 mediates chloride ion transport to inhibit the formation of NLRP3 inflammasome in macrophages.^[^ ^38^ ^]^ Consequently, we investigated whether bilirubin targeting WNK1 is involved in its anti‐inflammatory mechanism by modulating NLRP3 in neurons. We employed two genetically modified mouse strains, biliverdin reductase (*Blvra*) knockout *Blvra^−/−^* and UDP glucuronosyltransferase family 1 member A1 (*Ugt1a1*) knockout *Ugt1a1^+/−^*, to mimic the low‐ and high‐bilirubin conditions, respectively. *Blvra^−/−^* mice lack the gene encoding biliverdin reductase A, an enzyme crucial for converting biliverdin to bilirubin, resulting in reduced serum bilirubin levels.^[^ ^39^ ^]^ Conversely, *Ugt1a1^+/−^* mice exhibit decreased activity of UDP‐glucuronosyltransferase 1A1, the enzyme responsible for bilirubin conjugation, leading to elevated serum bilirubin levels.^[^ ^40^ ^]^ And we confirmed that bilirubin levels in the serum and hippocampal tissues of *Blvra^−/−^* and *Ugt1a1^+/−^* mice met the expected criteria (Figure S17, Supporting Information). Moreover, *Ugt1a1^+/−^* mice exhibited reduced KCC2 phosphorylation in the hippocampus, whereas *Blvra^−/−^* mice showed increased KCC2 phosphorylation (Figure S18, Supporting Information).

**Figure 5:**
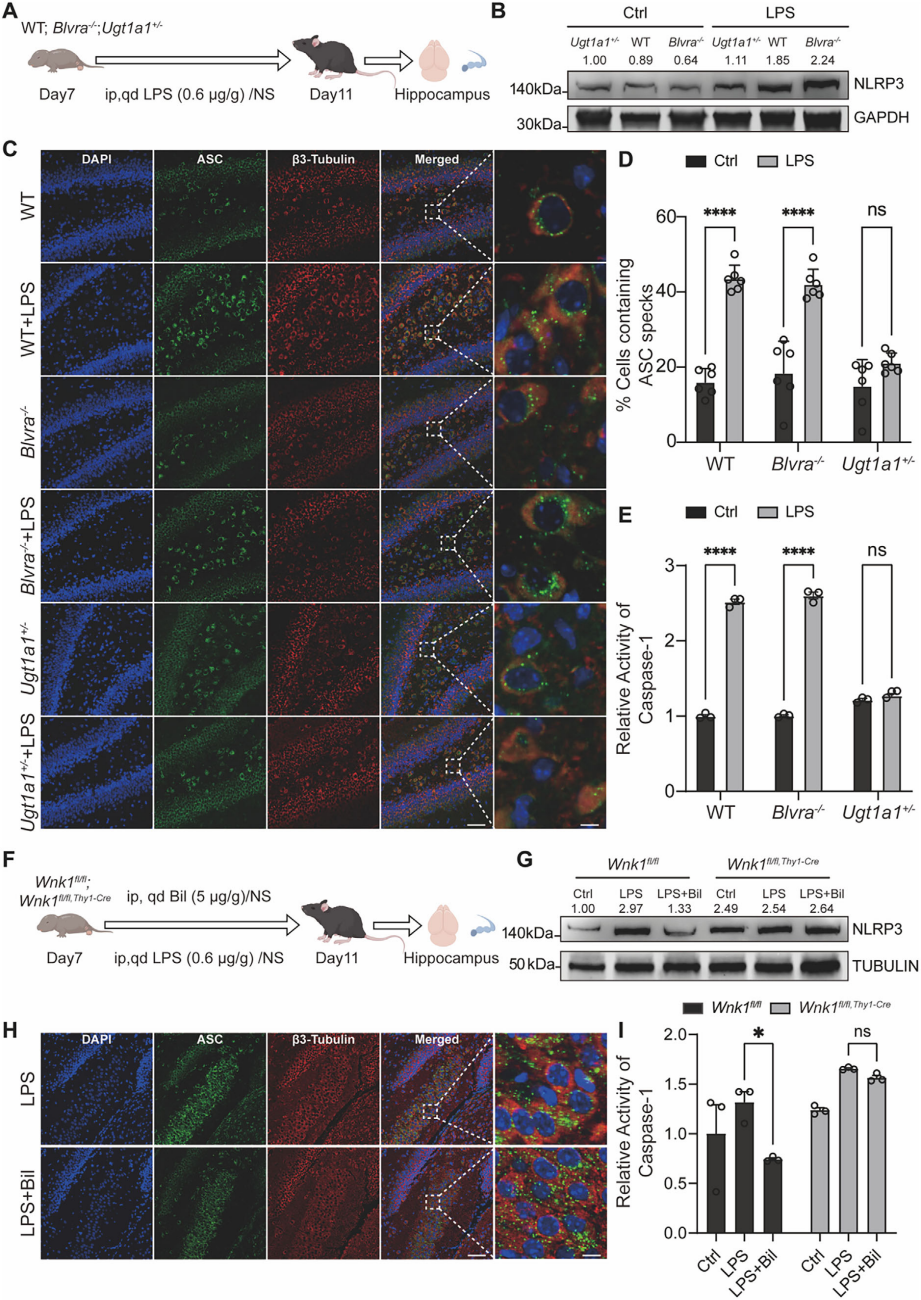
Bilirubin‐WNK1 suppresses LPS‐induced NLRP3 activation in the mouse hippocampus. A) Diagram of the experimental design. LPS (0.6 µg g^−1^) or normal saline (NS) was injected intraperitoneally (i.p.) for 5 consecutive days starting on day 7. Hippocampal tissues were harvested 6 h after last injection. B) Western blotting analysis of NLRP3 in hippocampal tissues from WT, *Blvra^−/−^*, or *Ugt1a1^+/−^* mice after LPS/saline treatment. C) Immunofluorescence staining of ASC speck formation in hippocampal tissues from WT, *Blvra^−/−^*, or *Ugt1a1^+/−^* mice after LPS/saline treatment. β3‐Tubulin was used to label neurons and DAPI to stain cell nuclei. Main image scale bars = 50 µm, inset scale bars = 5 µm. D) Summary data showing the percentage of cells containing ASC specks as shown in C) (*n* = 6). (E) Relative activity of caspase‐1 in hippocampal tissues from WT, *Blvra^−/−^*, or *Ugt1a1^+/−^* mice after LPS/saline treatment (*n* = 3). F) Experimental design diagram. LPS (0.6 µg g^−1^), LPS combined with bilirubin (5 µg g^−1^), or normal saline (NS) was injected intraperitoneally for 5 consecutive days starting on day 7. G) Western blotting analysis of NLRP3 in hippocampal tissues from *Wnk1^fl/fl,Thy1‐Cre^* mice after treatment. H) Immunofluorescence staining of ASC speck formation in hippocampal tissues from vehicle‐treated and *Wnk1^fl/fl,Thy1‐Cre^* mice after treatment. β3‐Tubulin was used to label neurons and DAPI to stain cell nuclei. Main image scale bars = 50 µm, inset scale bars = 5 µm. Corresponding images are also provided in Figure S21 (Supporting Information). I) Relative activity of caspase‐1 in hippocampal tissues from *Wnk1^fl/fl^* and *Wnk1^fl/fl,Thy1‐Cre^* mice after treatment (*n* = 3). Error bars indicate mean ± SD. *p < 0.05, **p < 0.01, ***p < 0.001, and ****p < 0.0001.

Furthermore, a murine model of infection‐induced inflammation was established in neonatal wild‐type, *Blvra^−/−^*, and *Ugt1a1^+/−^* mice by intraperitoneal administration of lipopolysaccharide (LPS) (**Figure** **5**A).^[^ ^41^, ^42^ ^]^ We assessed the expression levels of NLRP3 in the hippocampal region and the aggregation of the adaptor protein ASC in hippocampal neurons to indicate the level of inflammasome activation.^[^ ^43^, ^44^ ^]^ We observed elevated NLRP3 expression (Figure 5B) and increased ASC aggregation in low‐bilirubin *Blvra^−/−^* mice compared to wild‐type controls, whereas these parameters were reversed in high‐bilirubin *Ugt1a1^+/−^* mice (Figure 5C,D). Additionally, Caspase‐1 enzyme activity decreased in high‐bilirubin mice (Figure 5E), supporting the inhibitory role of bilirubin in NLRP3 activation.

To investigate the role of WNK1 in mediating the inhibitory effect of bilirubin on NLRP3 activation, we used *Wnk1^fl/fl,Thy1‐Cre^* mice which is the hippocampal neuron‐specific *Wnk1* knockout mice. Mice were then administered intraperitoneal injections of saline, LPS, or a combination of LPS and bilirubin (Figure 5F). After five days of LPS treatment, we observed increased staining areas for GFAP (an astrocyte marker) and IBA1 (a microglia marker). Furthermore, a portion of the adaptor protein ASC (encoded by PYCARD) co‐localized with IBA1 and GFAP. However, bilirubin does not affect ASC aggregation in either microglia or astrocytes (Figures S19 and S20, Supporting Information). Western blotting analysis and Caspase‐1 enzyme activity assays revealed that under LPS stimulation, the deletion of *Wnk1* abolished the inhibitory effect of bilirubin on NLRP3 inflammasome assembly, Caspase‐1 enzyme activity, as well as the cleavage‐mediated maturation of interleukin‐1β (IL‐1β) (Figure 5G–I; Figures S21 and S22A, Supporting Information). In contrast, neither bilirubin nor *Wnk1* deficiency had a significant effect on the phosphorylation‐mediated activation of the NF‐κB p65 subunit upon LPS stimulation (Figures S22B, Supporting Information). Building on the observation that bilirubin reduces Caspase‐1 activation, IL‐1β cleavage, and ASC speck formation, which are key markers of NLRP3 inflammasome activation, our results underscore that bilirubin primarily inhibits the activation phase of the NLRP3 inflammasome. In conclusion, these findings demonstrate that bilirubin binding to WNK1 effectively suppresses NLRP3‐mediated neuroinflammation.

### 2.6 Phosphoproteomic Analysis Reveals Potential Downstream Signaling Pathways of Bilirubin WNK1 Binding

**Figure 6:**
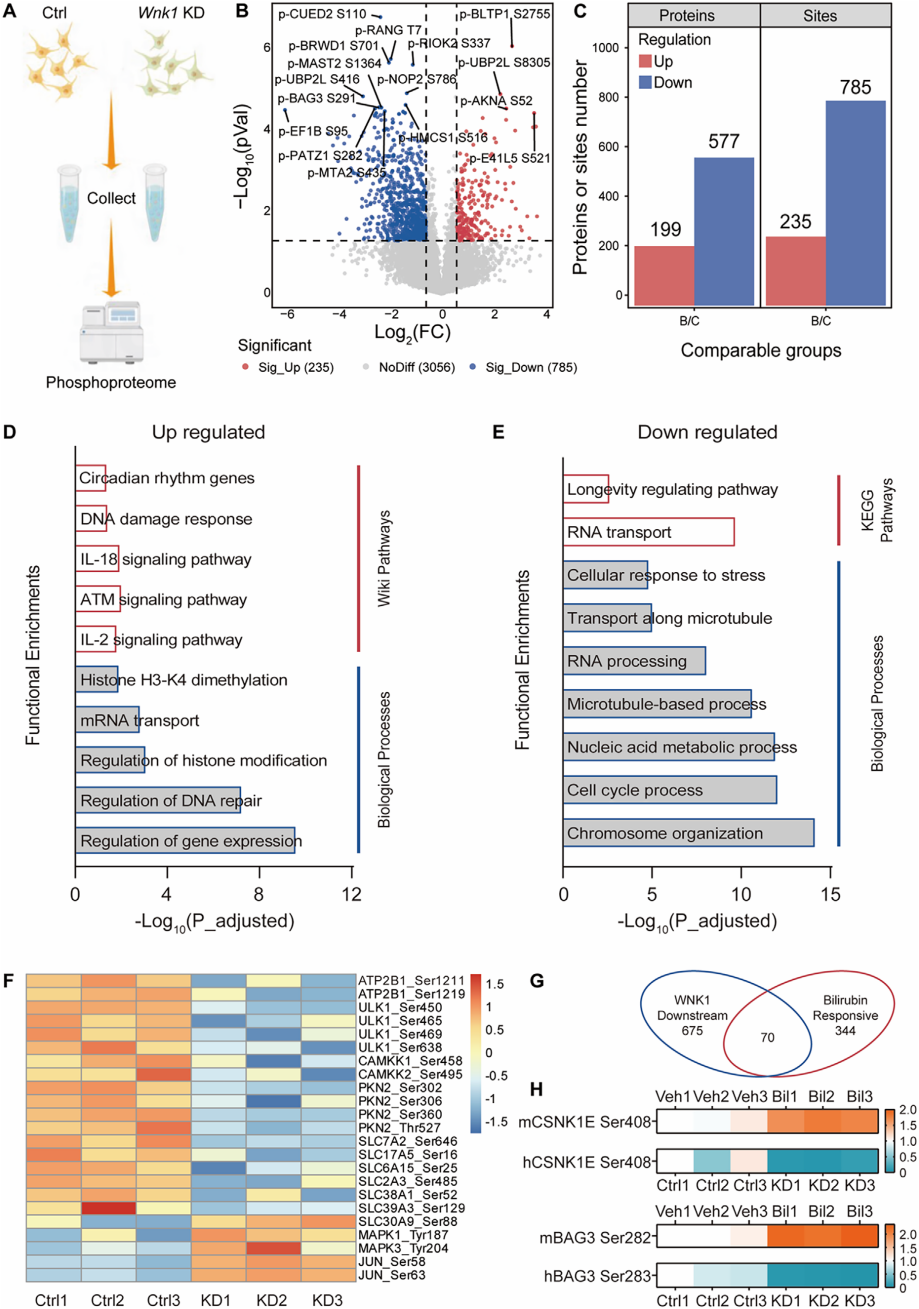
Phosphoproteomic analysis reveals WNK1 downstream signaling pathways. A) Diagram of the experimental design. SH‐SY5Y cells were subjected to WNK1 knockdown using shRNA. Both normal and WNK1 knockdown SH‐SY5Y cells were collected for quantitative phosphoproteomics analysis. B) Volcano plot illustrating identified phosphorylation sites after WNK1 knockdown by shRNA in SH‐SY5Y cells. C) Number of significantly regulated proteins and phosphorylation sites following WNK1 knockdown. D) Wiki Pathways (red) and biological processes (blue) in Gene Ontology enrichment analysis of proteins whose phosphorylation was upregulated after WNK1 knockdown. E) KEGG pathway analysis (red) and biological process (blue) in Gene Ontology enrichment analysis of proteins whose phosphorylation was downregulated after WNK1 knockdown. F) Overall phosphorylation change (−log_10_(*p*‐value)) of a protein complex related to WNK1 downstream function, estimated as the change in phosphorylation on member proteins. G) The phosphorylation levels of 70 proteins were significantly altered in both WNK1 downstream and bilirubin‐responsive omics. H) CSNK1E and BAG3 among 70 proteins in G) show opposite changes in phosphorylation levels in the two omics.

Apart from inflammation regulation, WNK1 signaling pathways are also involved in angiogenesis, cancer proliferation, metastasis, and other functions.^[^ ^17^, ^33^ ^]^ Therefore, WNK1‐mediated kinase signaling was further explored to gain a deeper understanding of the biological functions of bilirubin‐WNK1 binding. We generated *Wnk1* specific knockdown in human SHSY‐5Y cells and performed a quantitative phosphoproteomic analysis (**Figure** **6**A). *Wnk1* knockdown resulted in significant alterations in the phosphorylation levels of 785 sites in 577 proteins (fold change <0.667, *p* < 0.05) and 235 sites in 199 proteins (fold change >1.5, *p* < 0.05) out of 6916 detected phosphorylation sites (Figure 6B,C), indicating that WNK1 has extensive downstream phosphorylation pathways. Enrichment analysis demonstrated that proteins with decreased phosphorylation levels were predominantly associated with biological processes such as “microtubule‐based processes,” “RNA processing,” and “cell cycle,” as well as other signaling pathways such as “RNA transport” and “longevity regulating pathway” (Figure 6E). Proteins with increased phosphorylation levels were enriched in biological processes such as “regulation of gene expression” and “mRNA export” and were associated with signaling pathways like “DNA damage response,” “circadian rhythm genes,” and “IL‐2 and IL‐18 signaling pathways” (Figure 6D), which correspond to the anti‐inflammatory effects of WNK1.

Although the phosphorylation of classical downstream targets of WNK1, such as NKCC1 (SLC12A1) (Thr212, Thr203/207) and NKCC2 (SLC12A2) (Thr105, Ser130), was not detected in the phosphoproteomic analysis of *Wnk1*‐deficient SHSY‐5Y cells, we identified a reduction in the phosphorylation levels of other significant proteins.^[^ ^31^ ^]^ Specifically, the plasma membrane calcium transporter ATP2B1 (Ser1211 and Ser1219), sodium‐coupled neutral amino acid transporter SLC38A1 (Ser52), and vesicular excitatory amino acid transporter SLC17A5 (Ser16) showed decreased phosphorylation upon *Wnk1* knockdown (Figure 6F). This suggests that WNK1 may affect cellular transmembrane substance transport through modification of these proteins. Importantly, the phosphorylation levels of critical nodes in the inflammatory signaling pathway, including Erk1 (Mitogen‐activated protein kinase 3, MAPK3) at Tyr204, Erk2 (Mitogen‐activated protein kinase 1, MAPK1) at Tyr187, and AP‐1 transcription factor c‐Jun (JUN) at Ser58 and Ser63, were significantly upregulated following WNK1 knockdown (Figure 6F). This indicates that WNK1 broadly inhibits the activation of the inflammatory signaling pathways.

To further explore the contribution of the WNK1‐mediated phosphorylation signaling network to the bilirubin signaling pathway, we integrated phosphoproteomic datasets that were influenced by bilirubin and *Wnk1* knockdown. The analysis revealed that regardless of the specific phosphorylation sites, 70 proteins were co‐regulated by bilirubin and WNK1 (Figure 6G). Further analysis of the specific phosphorylation sites of these proteins revealed that only a few sites are simultaneously regulated by WNK1 and bilirubin. For example, phosphorylation of casein kinase CSNK1E at Ser408 and Bcl2‐associated athanogene BAG3 at Ser282 (corresponding to Ser283 in humans) increased after bilirubin stimulation, but decreased after *Wnk1* knockdown (Figure 6H), suggesting that they may be downstream targets of the bilirubin‐WNK1 signaling pathway. Overall, these results suggest that bilirubin‐WNK1 binding induces a variety of important cellular biological effects, including anti‐inflammatory effects, and reveals the potential downstream molecular mechanisms of WNK1.

## 3 Discussion

In recent years, bilirubin has garnered increased attention because of its diverse physiological functions. Importantly, bilirubin exhibits broad anti‐inflammatory effects and has shown significant potential for the treatment of inflammatory diseases.^[^ ^4^, ^5^ ^]^ Understanding the mechanism of bilirubin's anti‐inflammatory effects is of great significance for advancing the development and application of bilirubin‐based nanomedicines. In this study, we identified WNK1 as a direct binding target of bilirubin, which mediates the SPAK/OSR1‐KCC2 pathway to elevate intracellular chloride ions and inhibit NLRP3 activation in neurons. Consistent with our results, bilirubin has been shown to mitigate alum‐induced peritonitis by inactivating the NLRP3 inflammasome.^[^ ^45^, ^46^ ^]^ WNK1 has been reported to be a negative regulator of the NLRP3 inflammasome in macrophages.^[^ ^38^ ^]^ Additionally, we found that the phosphorylation levels of critical nodes in the inflammatory signaling pathway, such as IL‐2 and IL‐18, were significantly altered following WNK1 knockdown. These results suggest that the binding of bilirubin to WNK1 may represent a broad mechanism by which bilirubin exerts its anti‐inflammatory effects. Given the role of sustained neuroinflammation and NLRP3 inflammasome hyperactivation in various neurodegenerative diseases such as Alzheimer's disease (AD), Parkinson's disease (PD), and multiple sclerosis (MS), this study underscores the therapeutic potential of bilirubin and its WNK1 target in these neuroinflammatory disease management.^[^ ^43^, ^45^, ^47^ ^]^ However, the specific roles and regulatory mechanisms of the proposed bilirubin‐WNK1 signaling pathway in different inflammatory diseases require further investigation.

The phosphoproteomic analysis showed that bilirubin reduces the phosphorylation levels of WNK1 at residues Thr58 (Thr60 in humans), Ser183, and Ser185. WNK1 Thr58 phosphorylation has been implicated in a positive correlation with its kinase activity; however, this relationship is still under debate. For instance, a study by Ke et al. suggests that WNK1 regulation of intracellular chloride ion concentration is independent of Thr58 phosphorylation. The compound AK6 reduces phosphorylation at Thr60 while concurrently increasing intracellular chloride ion concentrations, indicating a potential negative correlation between Thr60 phosphorylation and WNK1 kinase activity.^[^ ^48^ ^]^ Additionally, reduced phosphorylation on S183 and S185 is not known to increase WNK1 kinase activity. This raises other possibility of regulatory mechanisms of bilirubin on WNK1 activity independent of these residues. As shown in Figure 4A, bilirubin treatment can directly activate the amino acid region 181–507 of hWNK1, suggesting that the active site of WNK1 can be modulated by bilirubin‐WNK1 binding, independent of upstream phosphorylation regulation. Small molecule activation of kinase function can occur through various mechanisms, including direct binding to the kinase active site, allosteric binding leading to conformational changes, or promotion of kinase oligomerization.^[^ ^49^ ^]^ Therefore, bilirubin may exert its effects through direct interaction with the kinase active site. Further exploration of the specific mechanism by which bilirubin activates the WNK1 kinase function will fill the gap in WNK1 agonist research and advance the biological applications of bilirubin as a therapeutic agent. Furthermore, we hypothesize that bilirubin binding may introduce a steric hindrance effect, preventing intracellular upstream kinases from phosphorylating critical residues such as Thr58, Ser183, and Ser185.

Bilirubin exerts a wide range of biological effects, including physiological actions such as anti‐inflammatory effects and metabolic regulation, as well as toxicological effects when present at excessive concentrations (i.e., hyperbilirubinemia), which can lead to neurotoxicity and pruritus. Several studies have identified multiple bilirubin targets with diverse binding affinities. FABP1 binds bilirubin at concentrations between 1–4 µm. Additionally, UnaG, a modified member of the FABP family derived from eel, has a high affinity for bilirubin (*Kd* = 98 pM) and is widely used as a tool for bilirubin detection.^[^ ^50^ ^]^ FABP1 and PPARα (*Kd* = 5.13 µM) in liver and adipose tissue, modulating various energy metabolic pathways,^[^ ^3^, ^51^ ^]^ while MRGPRs (*Kd* = 54.4–92.9 µM) in peripheral sensory neurons mediate itch sensation.^[^ ^3^, ^9^ ^]^ Additionally, bilirubin interacts with TRPM2 channels (EC50 = 1.9 µM), exacerbating Ca^2^⁺‐dependent brain injury during stroke.^[^ ^10^ ^]^ In comparison, our study found that bilirubin binds strongly to WNK1, with a binding affinity across a broader concentration range (*Kd* = 67.2 nM), which is relevant to both its anti‐inflammatory effects at physiological concentrations and its neurotoxic effects at higher concentrations in the central nervous system. These results suggest that WNK1 may play a crucial role in mediating bilirubin's dual effects, which depend on its concentration, tissue specificity, and physiological conditions. WNK1 is an intracellular serine/threonine kinase involved in regulating various cellular functions. While we did not find direct evidence linking WNK1 to other reported peripheral targets of bilirubin, this remains an interesting avenue for future research.

As we have stated above, our previous research demonstrated that acute ischemic stroke triggers a transient increase in bilirubin levels (>25 µM) in the region surrounding the injury, where bilirubin acts as a volume neurotransmitter to activate TRPM2 channels (EC50 = 1.9 µM), exacerbating Ca^2^⁺‐dependent excitotoxicity in the brain. TRPM2 is a highly Ca^2^⁺‐permeable, non‐selective cation channel expressed in immune cells and neurons, mediating Ca^2^⁺ influx that initiates signaling cascades responsible for neuronal excitability and injury associated with hyperbilirubinemia pathology. In this study, we discovered that bilirubin binds to WNK1 with a high binding affinity at low concentrations (*Kd* = 67.2 nM), exerting anti‐inflammatory effects in neurons. WNK1, an intracellular serine/threonine protein kinase, regulates ion transport (Na⁺, K⁺, Cl⁻) through the downstream kinases SPAK/OSR1, which maintains ion homeostasis. Our results suggested that the active site of WNK1 can be directly modulated by bilirubin‐WNK1 binding, independent of upstream phosphorylation regulation such as that mediated by TRPM2. Besides, it is important to note that bilirubin is not the specific ligand for TRPM2. Structural derivatives of bilirubin, such as biliverdin, can activate TRPM2 channels. In contrast, WNK1 can bind bilirubin but not biliverdin, indicating that WNK1 is a highly selective target of bilirubin in the central nervous system.

Moreover, astrocytes can exert either neuroprotective or neurotoxic effects depending on the microenvironment. Astrocytes engage in complex crosstalk with neurons in response to bilirubin. Studies have shown that bilirubin can activate the NLRP3 inflammasome and MAPK pathways in astrocytes in vitro, and it can also induce autophagy via mTOR/PKC/calcium signaling.^[^ ^52^ ^]^ Compared to astrocytes, neurons are more vulnerable to bilirubin exposure. Bilirubin influences various stages of neural circuit development, enhances synaptic transmission, and activates multiple ion channels, including TRPM2 and acid‐sensing ion channels, thus increasing neuronal excitability. In this study, we demonstrated that bilirubin binds to WNK1 at physiological concentrations, stimulating WNK1 kinase activity and suppressing LPS‐induced NLRP3 inflammasome activation. While both neurons and astrocytes can mount NLRP3 inflammasome responses, we observed that bilirubin‐induced changes in intracellular chloride concentration occur in hippocampal neurons but not in astrocytes, which lack KCC2 expression. These findings suggest that the bilirubin‐WNK1‐KCC2 pathway may represent a neuron‐specific signaling mechanism mediating the anti‐inflammatory effects of bilirubin in the hippocampus.

In addition to its role in inflammation modulation, bilirubin also plays a role in blood pressure regulation, although the exact mechanisms are not fully understood.^[^ ^3^, ^8^ ^]^ Previous studies have identified WNK1 as a risk gene for salt‐sensitive hypertension, as it regulates the activity of the Na^+^–Cl^−^ cotransporter (NCC) responsible for NaCl reabsorption in the distal convoluted tubule via the WNK1‐SPAK/OSR1 signaling cascade, which may be critically important in the development of hypertension.^[^ ^16^ ^]^ Our findings suggest that bilirubin binding to WNK1 may be a novel and essential mechanism underlying its role in blood pressure regulation. Furthermore, phosphorylation enrichment analysis suggested that casein kinase CSNK1E and BAG3 may be downstream targets of the bilirubin‐WNK1 signaling pathway. Given the roles of CSNK1E and BAG3 in circadian rhythm regulation and the rhythmic fluctuations in the expression and phosphorylation levels of WNK1,^[^ ^53^, ^54^ ^]^ we hypothesize that the bilirubin‐WNK1 pathway may play a regulatory role in rhythmic processes.

In conclusion, this study integrates phosphoproteomics with advanced molecular‐protein interaction techniques to identify WNK1 as a key target for bilirubin at physiological concentrations. Our results demonstrate that bilirubin binds to the kinase domain of WNK1, enhancing its activity and leading to the phosphorylation of downstream SPAK/OSR1, which in turn inhibits KCC2 activity and elevates intracellular chloride levels in neurons. Additionally, we employed an inflammation model to show that bilirubin binding to WNK1 effectively suppressed LPS‐induced NLRP3 inflammasome activation, thereby exerting anti‐inflammatory effects in mouse neurons. While WNK1 is highly conserved across multiple species, further studies involving human tissues or more clinically relevant models will be essential to validate the translational relevance and broader applicability of our findings. Moreover, the phosphoproteomic analysis of downstream signaling pathways of WNK1 has provided valuable insights into its diverse biological roles, particularly in the regulation of inflammatory pathways. Our findings establish WNK1 as a direct intracellular target of bilirubin's anti‐inflammatory effects, offering new perspectives on the physiological roles of bilirubin and suggesting potential therapeutic applications.

## 4 Experimental Section

### Animal and Ethical Approval

All experimental procedures were conducted in accordance with the Institutional Guidelines for Animal Care and Use and approved by the Institutional Animal Care and Use Committee of Shanghai Sixth People's Hospital, affiliated with Shanghai Jiao Tong University School of Medicine (Approval No:2020‐0285). Efforts were made to minimize animal discomfort and suffering throughout the study period. *Blvra^−/−^*, *Ugt1a1^+/−^ and Wnk1^flox/flox^* C57BL/6 mice containing loxP sites flanking exon 2 were generated by Cyagen Biosciences, Inc. (Suzhou, China). The *Wnk1^flox/flox^* C57BL/6 mice were bred with Thy1Cre transgenic mice to generate conditional *Wnk1* knockout mice. Animals were housed under specific‐pathogen‐free (SPF) conditions with controlled temperature and a 12‐hour light‐dark cycle and were provided with standard food and water ad libitum.

### TMT‐Tag Quantitative Phosphorylation Proteomics

C57BL/6 mice at postnatal day 10 (P10) were euthanized on ice, and their cochlear nuclei were dissected and incubated in artificial cerebrospinal fluid (ACSF), with or without 3 µM bilirubin, followed by rapid freezing in liquid nitrogen. The ACSF composition was 124 mM NaCl, 5 mM KCl, 1.2 mM KH_2_PO_4_, 1.3 mM MgCl_2_, 2.4 mM CaCl_2_, 24 mM NaHCO_3,_ and 10 mM glucose (pH 7.4).

Tissue samples were ground in liquid nitrogen, lysed in urea buffer with protease inhibitors, sonicated, and centrifuged. Protein concentration was determined using the BCA assay. Trypsin digestion was performed, followed by TMT labeling, according to the manufacturer's protocol. Tryptic peptides were fractionated using high‐pH reverse‐phase HPLC and phosphopeptide enrichment was conducted using IMAC microspheres. LC‐MS/MS analysis was performed using a Q Exactive Plus instrument, with MS/MS data searched against a specified database using MaxQuant, employing predefined parameters for enzyme specificity, mass tolerances, modifications, and false discovery rate thresholds.

### Bioinformatics Analysis

Phosphoproteomic analysis identified 13495 phosphorylation sites distributed across 3792 proteins, with 11116 sites on 3474 proteins containing quantitative information. To ensure data reliability, we filtered the identified sites using a localization probability threshold of >0.75. After filtering, 10246 phosphorylation sites on 3540 proteins were retained, including 9404 sites on 3325 proteins with quantitative information. This dataset was subsequently used for bioinformatics analysis.

Differential phosphorylation sites were identified based on a fold‐change threshold of 1.5 and a t‐test *p*‐value < 0.05. Using these criteria, we found that 126 phosphorylation sites exhibited increased modification levels, and 469 sites showed decreased modification levels in the B/C (Bilirubin Treated/Control) comparison group.

Proteins with quantitative information were subjected to systematic bioinformatics analysis, including protein annotation, functional classification, functional enrichment analysis, and cluster analysis based on enrichment results.

For the quantification of significantly differential phosphorylation, each phosphorylation site was analyzed in three biological replicates. The average quantitative value for each site across three replicates was calculated for both groups. The ratio of the average values between the two groups was used as the relative quantification of differential modification (Ratio). Quantitative values were log2‐transformed to approximate normal distribution, and statistical significance was evaluated using a two‐tailed t‐test. A *p*‐value < 0.05 combined with a fold change >1.5 was considered as a significant increase, whereas a fold change <1/1.5 was considered as a significant decrease.

For the assessment of quantification reproducibility, the reproducibility was evaluated using principal component analysis (PCA), relative standard deviation (RSD), and Pearson's correlation coefficient across biological replicates.

### Plasmid Construction

All WNK1 clones were derived from the original untagged hWNK1 cDNA (NM_018979), which was modified and subcloned into the pcDNA3.1, referred to as “full‐length WNK1” throughout this study. Truncated constructs, including 1–190aa, 191–491aa, 1–491aa, and 491–2382aa fragments, were generated by PCR using the full‐length template and subsequently ligated into linearized pcDNA3.1 vectors. WNK2 was obtained from Addgene Plasmid #24569, and WNK3 was obtained from Addgene Plasmid #81723. Both were subjected to base modifications and cloned into pcDNA3.1. The WNK4 clone was derived from hWNK4 cDNA (NM_032387.5), modified, and subcloned into the pCMV3‐C‐GFPSpark vector.

### Cell Culture and Transfection

Human embryonic kidney cells (HEK‐293T, ATCC: CRL‐11268) and SH‐SY5Y cells (ATCC: CRL‐2266) were cultured in Dulbecco's Modified Eagle Medium (DMEM, cat. no. 52100–39, Thermo Fisher Scientific) supplemented with 10% fetal bovine serum (FBS, cat. no. F7524, lot no. 022M3395, Sigma‐Aldrich) and 1% (v/v) streptomycin/penicillin (cat. no. L0022; Biowest) in a humidified atmosphere containing 5% CO₂ at 37 °C. Plasmid transfection was performed using Lipofectamine 3000 Transfection Reagent (cat. no. L3000075, Thermo Fisher Scientific). After transfection, the culture medium was replaced with a complete medium. To ensure consistency, the cells were characterized after one passage in the same culture medium to confirm maintenance of the desired characteristics and functionality.

### Wnk1 Knockdown Stable Cell Line Generation

Lentiviral constructs harboring short hairpin RNAs (shRNAs) targeting h*Wnk1* were used for the knockdown. The sequences used were 1‐GCTGCGTATTGAAGATATTAA and 2‐CGTCAGTATCAGTCCCTATAA. The pLKO.1 lentiviral expression vector containing the puromycin resistance gene was used to co‐express individual shRNAs. Recombinant lentiviral particles were generated by the transient transfection of plasmids into HEK‐293T cells. Stably transfected SH‐SY5Y cells were established using the lentiviral constructs. Following viral infection for 24 h, the medium was replaced with a complete medium supplemented with 2 µg mL^−1^ puromycin. After an additional 24‐hour incubation period with fresh puromycin‐containing medium, surviving cells were collected, expanded, and assessed for knockdown efficiency.

### Chemical Structures

Chemical structures were drawn using *ChemDraw* software.

### Western Blotting

Cells or tissues were lysed by grinding in RIPA buffer (cat. no. PC101; Epizyme Biotech) supplemented with a protease inhibitor cocktail (cat. no. 5872S; Cell Signaling Technology) at 4 °C. The lysate was subsequently centrifuged at 16000 × *g* for 30 min at 4 °C and the resulting supernatant was collected. The total protein concentration was determined using the Pierce BCA Protein Assay Kit (cat. no. 23 225, Thermo Fisher Scientific). The samples were denatured in a Protein Sample Loading Buffer (cat. no. LT101; Epizyme Biotech) at 95 °C and subjected to SDS‐polyacrylamide gel electrophoresis. Following electrophoresis, the proteins were transferred onto a nitrocellulose membrane (cat. no. HATF00010; Millipore) using a transfer buffer composed of 25 mM Tris, 190 mM glycine, and 20% methanol. Membranes were blocked with blocking buffer for western blotting (cat. no. P0252; Beyotime Biotechnology) at room temperature for 15 min, followed by overnight incubation with the primary antibody at 4 °C. After primary antibody incubation, the membranes were washed and incubated with secondary antibody at room temperature for 1 h. Protein bands were visualized using a Bio‐Rad chemiluminescence detection system upon the addition of a chemiluminescent substrate (Pierce ECL Western Blotting Substrate, cat. no. 32 106, Thermo Fisher Scientific).

### Immunofluorescence

The mice were anesthetized with 1% pentobarbital (0.04 mL/10 g) before the sternum was cut to expose the heart. A needle was inserted into the left ventricle to perfuse the pre‐oxygenated cold ACSF with low‐molecular‐weight heparin. Perfusion was continued until the tail, limbs, and liver turned pale (while the heart was kept beating), at which point the solution was switched to lavage buffer containing 4% paraformaldehyde. The brain tissue was fully fixed by perfusion for ≈15 min. Nuclear staining was performed using 4′,6‐diamidino‐2‐phenylindole (DAPI) (2 mg mL^−1^, Sigma).

### Molecular Docking and Virtual Screening

The crystal structure of WNK1 (PDB ID: 5TF9) was retrieved from the Protein Data Bank. Molecular docking studies were conducted using the Schrödinger Maestro software suite (Schrödinger, 2020–3). The protein was prepared using the Protein Preparation Wizard to address missing residues, remove water molecules, and optimize hydrogen bonding and energy using the OPLS2005 force field with a 0.30 Å RMSD.

The Receptor Grid Generation module was employed to define the grid files. Grid file 1 (site 1) was generated with ANP at the center to represent the ATP‐binding pocket, whereas grid file 2 (site 2) was centered around ligand molecule 7AV to represent the ligand‐binding pocket. Additionally, the Sitemap module was used to predict the binding pocket of hWNK1. Grid files 3–5 (sites 3–5) were generated using VAL318, TYR420, and SER469.

The 2D structure of bilirubin (C33H36N4O6) was subjected to energy minimization using the LigPrep module (OPLS2005 force field, 0.30 Å RMSD) to generate the corresponding 3D structure. Molecular docking was then performed using the Glide module in high‐precision (XP) mode, facilitating the docking of receptor and ligand molecules through geometric and energy matching.

Further molecular docking and virtual screening of bilirubin, which has a potential binding affinity to WNK1, were conducted using Guangzhou Yinfo Information Technology. The structural prediction of WNK1 was accomplished using the AlphaFold multimer in ColabFold, while the potential binding sites of WNK1 were predicted using the online tool POCASA 1.1. Molecular docking‐based virtual screening between WNK1 and bilirubin was performed using DOCK 6.9.

### MQAE Intracellular Cl⁻ Concentration Measurements

Intracellular chloride concentration ([Cl⁻]_i_) was measured using the MQAE fluorescent dye (Life Technologies) following established protocols (Zhang, Lee et al. 2015). Cultured primary hippocampal neurons were loaded by incubating cells in artificial cerebrospinal fluid (ACSF) containing 5 mM MQAE for 30 min at 37 °C. Imaging was subsequently performed using Zeiss confocal microscopy.

### Primary Neuronal Culture

Hippocampal neurons were isolated from C57BL/6 embryonic day 18 (E18) embryos, as previously described (Lee, Abiraman et al. 2021). Briefly, neurons were plated at a density of 5 × 10⁶ cells per dish and cultured in a neurobasal medium supplemented with 2% B27, 0.6% glucose, 1% GlutaMAX, and 0.5% penicillin/streptomycin. Cultures were maintained at 37 °C in a humidified atmosphere containing 5% CO₂ until further experimentation. The neuronal cell culture reagents were purchased from Invitrogen.

### Microscale Thermophoresis (MST)

In HEK293T cells, proteins tagged with green fluorescent protein (GFP) were expressed. The cells were lysed using the M‐PER Mammalian Protein Extraction Reagent (cat. no. 78505, Thermo Scientific) supplemented with protease inhibitors. Lysates were centrifuged at 12000 × g for 15 min at 4 °C, and the supernatant containing proteins was diluted 10‐fold with 1x PBST. Protein fluorescence intensity and solubility were assessed using capillary scanning at 60% LED power.

For the binding assay, the proteins were mixed with ligands and incubated for 15 min at room temperature before being loaded into Monolith NT.115 Capillaries or NT.115 Premium Capillaries (NanoTemper Technologies, MO‐K022 and MO‐K025). MST measurements were performed on a Monolith NT.115 instrument (NanoTemper Technologies) at 25 °C. Instrument parameters included no more than 80% excitation power and a medium MST power. Data analysis was conducted using the MO. Affinity Analysis software (NanoTemper Technologies).

### MS‐CETSA Sample Preparation

Cellular Thermal Shift Assay (CETSA) was performed following established procedures.^[^ ^21^, ^55^ ^]^ Briefly, SH‐SY5Y cells were treated with a serum‐free culture medium containing 6 µM bilirubin or an equivalent amount of DMSO for 30 min. After three washes with PBS, cells were collected and subjected to a 3‐minute heat shock at 55 °C, followed by immediate cooling on ice. The cells were lysed by repeated freeze‐thaw cycles in liquid nitrogen, and lysates were centrifuged at 20000 × *g* for 20 min at 4 °C to separate soluble proteins from denatured precipitated proteins. The supernatant containing the soluble proteins was collected for subsequent mass spectrometry analysis.

### LC‐MS Analysis

For peptide labeling, iTRAQ labeling reagent was used according to the number of samples. Briefly, peptide samples were dissolved in 0.5 m TEAB and added to the iTRAQ reagents, then left at room temperature for 2 h. Then, peptide fractionation was performed using a Shimadzu LC‐20AB system with a 5 µm 4.6 × 250 mm Gemini C18 column. Dried peptide samples were reconstituted with mobile phase A (5% ACN, pH 9.8), injected, and eluted using a gradient of mobile phase B (95% ACN, pH 9.8). Peaks were monitored at 214 nm, collected into 20 fractions, and freeze‐dried. The reconstituted samples were centrifuged and injected into a Thermo UltiMate 3000 UHPLC system. The peptides were ionized using a nanoESI source and analyzed on a Q‐Exactive HF X mass spectrometer (Thermo Fisher Scientific) in the DDA mode. MS data were acquired using a top 20 data‐dependent acquisition protocol at 60000 resolutions in MS1 and 15000 resolutions in MS2.

### Kinase Activity Measurements

WNK1 kinase activity was measured using the ADP‐Glo Kinase Assay (Promega, Cat. V9101) following the manufacturer's instructions. Purified protein was incubated with bilirubin across a range of 12 serially diluted concentrations, beginning at a maximum concentration of 20 µm. After the incubation period, luminescence was recorded using a microplate reader (Synergy H1M, BioTek)

### Caspase‐1 Activity Measurements

Caspase‐1 activity was measured using a Caspase‐1 Activity Assay Kit (Beyotime Biotechnology, Haimen, China). Briefly, 50 µg of protein from the mouse hippocampal tissue was added to a reaction buffer containing Ac‐YVAD‐ρNA. The absorbance was read at 405 nm using a microplate reader, and caspase‐1 activity was normalized to that of the control.

### Measurement of Unbound Bilirubin Using UnaG Fluorescence

To establish a standard calibration curve, reaction mixtures were prepared containing 50 µL of UnaG solution (1 µm) and 50 µL of bilirubin solutions at graded concentrations (1.4278, 0.7139, 0.3573, 0.1785, 0.0893, and 0 µm). After a 10‐minute incubation, fluorescence intensity was measured with a microplate reader (Synergy H1M, BioTek) at excitation and emission wavelengths of 485 and 528 nm. Mouse serum samples were diluted and incubated directly with UnaG, while hippocampal samples were homogenized in native lysis buffer (Beyotime, Cat.No.: P0013), centrifuged, and the supernatant incubated with UnaG. UCB levels were extrapolated from the standard curve, and hippocampal UCB concentrations were normalized to protein content and compared to wild‐type mouse levels.

### Statistical Analysis

Generally, all continuous variables were analyzed using parametric tests (two‐tailed unpaired T‐test assuming equal variances when comparing 2 groups, or one‐way ANOVA and post‐hoc Tukey LSD tests for >2 group comparisons). For proteomic analyses, we used un‐adjusted p values + log2(FC) thresholds to prioritize differentially abundant proteins. Power calculations were not performed for individual experiments. Specific statistical tests used for individual experiments are detailed in the figure legends. Sample size (*n*) for each statistical analysis and data presentation (mean ± SD) are provided in the figure legends. All statistical analyses were performed using GraphPad Prism 10.

## Conflict of Interest

The authors declare no conflict of interest.

## Author Contributions

L.M., J.L., Q.Y., and Z.L. contributed equally to this work. S.Y., C.L., and F.L. conceptualized the study; G.Y., R.S., J.L., and Q.Y. provided conceptual guidance; methodology was developed by F.L., L.M., Z.L., Q.Y., J.L., K.S., and Y.C; investigation was conducted by L.M., Z.L., B.K., Y.W., and Y.C; data curation was performed by L.M. and F.L.; formal analysis was carried out by F.L.; visualization was executed by C.W., H.Y., and K.S.; resources were provided by S.Y., L.M., J.L., C.L., and Q.Y.; funding acquisition was managed by C.L., S.Y., and G.Y.; project administration was led by S.Y. and C.L., with coordination by G.Y.; supervision was provided by S.Y., C.L., and G.Y.; L.M., F.L., and C.L. wrote the original draft and prepared it; F.L., C.L., J.L., Q.Y., and R.S wrote te original draft and edited the original manuscript and reviewed it.

## Supporting information



Supporting Information

## Data Availability

The data that support the findings of this study are available on request from the corresponding author. The data are not publicly available due to privacy or ethical restrictions.
