# Supplementary material for: Bilirubin Targeting WNK1 to Alleviate NLRP3‐Mediated Neuroinflammation
Source: Adv Sci (Weinh). 2025 Mar 20;12(29):2407349. doi: 10.1002/advs.202407349 (PMC12362796; doi:10.1002/advs.202407349)
Supplement: Supplementary file 1 — Supporting Information [file ADVS-12-2407349-s001.docx]

Supporting Information

Bilirubin Targeting WNK1 to Alleviate NLRP3-Mediated Neuroinflammation

Linfei Mao, Jiayu Lu, Quanjun Yang, Zhenqi Liu, Cuiping Wu, Bingbing Ke, Haolin Yuan, Yaqi Cui, Yao Wang, Richard Salvi, Guang Yang*, Shankai Yin, Feng Liu*, Chunyan Li*

This file includes:

Supplementary Figure S1-S23 and Table 1

**Figure S1. Phosphorylation Analysis and Kinase Regulation Following Bilirubin Treatment.** (A) Principal component analysis (PCA) of phosphorylation replicates after removing outliers. (B) Heatmap of Pearson correlation coefficients for all quantified proteins between each pair of samples. (C) Box plot of the relative standard deviation (RSD) distribution for quantified proteins within a biological condition (green) and bilirubin treatment conditions (red). Box plots depict median (horizontal lines), interquartile range (boxes), maximum and minimum values (vertical lines), and outliers (solid circles). (D) Gene Ontology enrichment analysis of all significantly changing proteins in response to bilirubin treatment, categorized into biological process (blue), molecular function (yellow), and cellular component (red). (E) Kyoto Encyclopedia of Genes and Genomes (KEGG) pathway analysis of proteins that are up-regulated (red) and down-regulated (green) after bilirubin treatment. (F) Kinases significantly regulated after bilirubin treatment, annotated to major kinase families using [www.kinhub.org](http://www.kinhub.org). TK, tyrosine kinases; TKL, tyrosine kinase-like; STE, homologs of yeast STE7, STE11, and STE20 genes; CK1, casein/cell kinase 1 family; AGC, protein kinase A, G, C families; CAMK, calmodulin/calcium-regulated kinases and some non–calcium-regulated families; CMGC, CDK, MAPK, GSK3, and CLK kinase families. Atypical kinases are shown as gray branches. (G) Phosphorylation sites of significantly regulated kinases after bilirubin treatment, with up-regulated sites in red and down-regulated sites in green.

**Figure S2. Motif Enrichment Analysis of Serine and Threonine Modification Sites.** (A) Motif enrichment heatmap of upstream and downstream amino acids for all identified serine modification sites. Red indicates significant enrichment near the modification site, and green indicates significant reduction. (B) Summary of modified site feature sequences and enrichment statistics from MoMo software. (C) Motif enrichment heatmap of upstream and downstream amino acids for all identified threonine modification sites. Red indicates significant enrichment near the modification site, and green indicates significant reduction.

**Figure S3. Differential Protein Levels Following Bilirubin Treatment.** (A) Volcano plot showing differential soluble protein levels between control and bilirubin-treated SH-SY5Y cells.

**Figure S4. Gene Ontology Enrichment Following Bilirubin Treatment** (A) Gene Ontology enrichment analysis of significantly up-regulated proteins in response to bilirubin treatment, categorized into biological process (BP), molecular function (MF), and cellular component (CC). (B) Gene Ontology enrichment analysis of significantly down-regulated proteins in response to bilirubin treatment, categorized into biological process (BP), molecular function (MF), and cellular component (CC).

**Figure S5. CETSA-MS and Expression Analysis of WNK1 in Response to Bilirubin Treatment.** (A) Volcano plot of CETSA-MS following bilirubin treatment. All bilirubin treatment groups were compared with control groups, and bilirubin potential binding proteins were defined as having an absolute value of bilirubin/control > 1, adjusted *p* < 0.05, and identified peptide count > 5. (B) Anatomogram illustrating WNK1 expression in human tissues from The Human Protein Atlas. (C) Heatmap showing the expression of WNK1 RNA in human brain regions sensitive and insensitive to bilirubin (from the Allen Brain Database). (D) Fluorescence microscope images showing the expression of WNK1 (green) and GAD67 (red) in the mouse cochlear nucleus. Scale bar = 50 µm. (E) Fluorescence microscope images showing the expression of WNK1 (red), Tau (green), and GFAP (pink) in the mouse hippocampus. Scale bar = 100 µm.

**Figure S6. Protein Domain Enrichment Analysis Following Bilirubin Treatment.** (A) Protein domain enrichment bubble plot for proteins corresponding to up-regulated phosphorylation sites after bilirubin treatment. (B) Protein domain enrichment bubble plot for proteins corresponding to down-regulated phosphorylation sites after bilirubin treatment.

**Figure S7. Phosphorylation Sites of WNK1 in Response to Bilirubin Treatment.** (A) Summary of significantly regulated phosphorylation sites of WNK1 after bilirubin treatment. (B-D) Identified phosphorylated peptide mass spectrum of p-WNK1 at Thr58, Ser183, and Ser185.

**Figure S8. Direct Interaction Analysis of Bilirubin with WNK2, WNK3, and WNK4 and Conservation Analysis of WNK1.** (A) MST demonstrating direct interaction between bilirubin and EGFP-tagged WNK2, WNK3, and GFP Spark-tagged WNK4 (15 min) in lysates from WNK2, WNK3, and WNK4 expressing HEK293T cells (n = 3/group). Error bars indicate mean ± SD. (B) Conservation analysis of hWNK1 sequence across different species. Sequence identity of different species' WNK1 is from the NCBI database and divided into three regions: the NTD, the kinase domain (STKc_WNK1), and the large C-terminal domain (CTD).

**Figure S9. Specific Recognition of Bilirubin-Bound Pockets in WNK1.** (A) Specific recognition of the bilirubin-bound hWNK1 Val318 pocket. (B) Specific recognition of the bilirubin-bound hWNK1 Tyr420 pocket. (C) Specific recognition of the bilirubin-bound hWNK1 Ser469 pocket.

**Figure S10.** Western blotting analysis of His-hWNK1 (181-507 S382A) using an anti-His Tag antibody, confirming the expression of the His-tagged WNK1 protein.

**Figure S11.** Osmolality measurements of ACSF solutions containing varying concentrations of bilirubin (n = 3). Error bars represent mean ± SD. Statistical analysis was performed using one-way ANOVA.

**Figure S12. WNK1 Inhibitor Suppresses Bilirubin-Induced Increase in Spontaneous Firing of Cochlear Nuclei Neurons.** (A) Spontaneous activity recorded from a cochlear nucleus neuron from P8 mice under control and bilirubin (9 μM) conditions. (B) Summary data of average spontaneous firing frequency from (A) (n = 6). (C) Spontaneous activity recorded from a cochlear nucleus neuron from P8 mice under control, WNK-IN-11 (1 μM), and WNK-IN-11 (1 μM) with bilirubin (9 μM) conditions. (D) Summary data of average spontaneous firing frequency from (C) (n = 6). Error bars indicate mean ± SD. **p* < 0.05, ***p* < 0.01, ****p* < 0.001, and *****p* < 0.0001.

**Figure S13. Bilirubin Enhances Excitability of Hippocampal Neurons.** (A) Representative current-clamp recordings showing neuronal firing in response to varying injected currents, before and after the application of 3 or 6 μM bilirubin. (B) Quantification of spike numbers at different injected currents, comparing neuronal firing before and after bilirubin perfusion (n = 6). (C) Measurement of input resistance at varying injected currents, before and after bilirubin perfusion (n = 6). Error bars represent mean ± SD. Statistical analysis was performed using one-way ANOVA.

**Figure S14. Bilirubin-Induced Changes in Intracellular Chloride Concentration in Neurons.** (A) Representative immunofluorescence staining results of bilirubin-induced [Cl⁻]_i_ changes in DIV8 primary cultured hippocampal neurons, with or without bumetanide, gabazine, VU-0463271, bicuculline and strychnine. MQAE staining (green) marks [Cl⁻]_i_ (fluorescence intensity inversely proportional to intracellular chloride concentration), and CellTracker staining (red) marks live cells. Scale bars = 50 μm.

**Figure S15. Bilirubin Has No Effect on Intracellular Chloride Concentration in Astrocytes and HEK 293T Cells.** (A) Representative immunofluorescence staining results of bilirubin-induced [Cl⁻]_i_ changes in primary cultured hippocampal astrocytes. MQAE staining (green) marks [Cl⁻]_i_ (fluorescence intensity inversely proportional to intracellular chloride concentration), and CellTracker staining (red) marks live cells. (B) Summary data showing the fold-change in the MQAE fluorescence intensity of astrocytes in (A). (C) Fold-change in the MQAE intensity of HEK 293T cells induced by bilirubin in normal or Cl⁻-free solution. Error bars indicate mean ± SD. **p* < 0.05, ***p* < 0.01, ****p* < 0.001, and *****p* < 0.0001.

**Figure S16. Expression of KCC2 in Neurons, Astrocytes, Microglia, and Oligodendrocytes.** (A) Immunofluorescence images showing the expression of KCC2 in various cell types, including neurons, astrocytes and microglia. Neurons were labeled with NEUN, astrocytes with S100β, and microglia with IBA1. Main image scale bars = 50 μm, inset scale bars = 5 μm. (B) Single-cell RNA sequencing data from the Allen Brain Atlas (10x scRNA-seq, whole brain), showing KCC2 mRNA expression across different cell populations.

**Figure S17.** PCR genotyping results for (A) *Ugt1a1^+/-^* and (B) *Blvra^-/-^* mice. (C, D) Relative levels of free bilirubin in serum (C) and hippocampal tissue (D) across wild-type (WT), *Ugt1a1^+/-^*, and *Blvra^-/-^* mice (n=3). Error bars represent mean ± SD. Statistical analysis was performed using one-way ANOVA. **p* < 0.05, ***p* < 0.01, ****p* < 0.001, and *****p* < 0.0001.

**Figure S18. Representat p-KCC2 in Hippocampal Tissue from *Blvra^-/-^*, Wild Type and *Ugt1a1^+/-^* mice.**

**Figure S19. Immunofluorescence images show the expression of ASC with GFAP (astrocytes) in hippocampal sections.** Samples were obtained from the brains of *Wnk1^fl/fl^ and Wnk1^fl/fl, Thy1-Cre^* mice, subjected to saline, LPS, or LPS with bilirubin treatments. Scale bars = 50 μm.

**Figure S20.** **Immunofluorescence images show the expression of ASC with IBA1 (microglia) in hippocampal sections.** Samples were obtained from the brains of *Wnk1^fl/fl^ and Wnk1^fl/fl, Thy1-Cre^* mice, subjected to saline, LPS, or LPS with bilirubin treatments. Scale bars = 50 μm.

**Figure S21. Immunofluorescence staining of ASC speck formation in hippocampal tissues from *Wnk1^fl/fl^* and *Wnk1^fl/fl, Thy1-Cre^* mice after treatment.** β3-Tubulin was used to label neurons and DAPI to stain cell nuclei. Main image scale bars = 50 μm, inset scale bars = 5 μm. Images of *Wnk1^fl/fl, Thy1-Cre^* mice treated with LPS, LPS and Bil are included in Figure 5H in the main text.

**Figure S22.** (A) pro-IL-1β and IL-1β, (B) p-p65 and p65 levels in hippocampal tissue from *Wnk1^fl/fl^* and *Wnk1^fl/fl, Thy1-Cre^* mice with saline, LPS and LPS with bilirubin. The inflammation model was established by intraperitoneal injection of saline, LPS (0.6 μg/g), or LPS (0.6 μg/g) with bilirubin (5 μg/g) once daily for 5 consecutive days, starting on postnatal day 7. Hippocampal tissues were collected 6 hours after the final injection for western blotting.

**Figure S23. The original images of western blotting.** (A) Original images corresponding to Figure 1F. (B) Original images corresponding to Figure 1G. (C) Original images corresponding to Figure 4B. (D) Original images corresponding to Figure 4G. (E) Original images corresponding to Figure 5B. (F) Original images corresponding to Figure 5G. (G) Original images corresponding to Figure S10A. (H) Original images corresponding to Figure S18A. (I) Original images corresponding to Figure S22A. (J) Original images corresponding to Figure S22B.

**Supplementary Table 1. Antibodies and chemicals information.** The working dilution for all antibodies were 1:1000 for western blotting and 1:200 for immunohistochemistry.
